# Anisotropic Memristive Switching in NbOCl_2_ Enabled by Directional Oxygen Ion Migration

**DOI:** 10.1002/advs.76461

**Published:** 2026-07-13

**Authors:** Caokun Wang, Yun Ji, Sanchali Mitra, Yufei Shi, Haofei Zheng, Yee Sin Ang, Kah‐Wee Ang

**Affiliations:** ^1^ Department of Electrical and Computer Engineering National University of Singapore 4 Engineering Drive 3 Singapore Singapore; ^2^ Science, Mathematics and Technology Singapore University of Technology and Design 9 Somapah Road Singapore Singapore

**Keywords:** anisotropy, lateral memristors, NbOCl_2_, oxidation, short‐term plasticity

## Abstract

Directional ion transport in anisotropic two‐dimensional (2D) materials provides a powerful yet underexplored pathway for engineering memristive functionalities. Here, we demonstrate strongly orientation‐dependent memristive switching in layered NbOCl_2_ lateral devices, where resistive switching occurs exclusively along the in‐plane c‐axis, while devices aligned along the b‐axis exhibit no hysteresis. The c‐axis devices show forming‐free volatile memristive behavior with a stable on/off ratio and cycle‐to‐cycle reproducibility. Combined experiments and first‐principles calculations reveal that the anisotropic switching originates from direction‐dependent oxygen‐ion migration and vacancy propagation, which modulate the Schottky barrier at the Pd/NbOCl_2_ interface. The resulting ion‐driven barrier modulation enables key synaptic functions, including excitatory postsynaptic current, paired‐pulse facilitation, spike‐rate‐dependent plasticity, and spike‐amplitude‐dependent plasticity, demonstrating short‐term synaptic plasticity. Leveraging the intrinsic nonlinear and self‐relaxation dynamics, the NbOCl_2_ memristor is further implemented as a physical reservoir, achieving 94.3% accuracy in handwritten digit recognition using the MNIST dataset. These results reveal NbOCl_2_ as a promising anisotropic memristive material and highlight directional ion migration in 2D systems as a versatile strategy for neuromorphic hardware and reservoir computing applications.

## Graphical abstract

This study investigates strongly orientation‐dependent memristive switching in anisotropic NbOCl_2_, observed exclusively along the in‐plane c‐axis. The switching originates from direction‐selective oxygen‐ion migration and vacancy propagation that modulate the Pd/NbOCl_2_ Schottky barrier, enabling short‐term plasticity. The device's intrinsic nonlinear and self‐relaxation dynamics further support reservoir computing with high‐accuracy handwritten digit recognition.

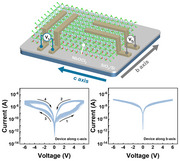

## 1 Introduction

To overcome the fundamental limitations of conventional von Neumann architectures, where memory and processing units are physically separated and data transfer becomes a major energy and latency bottleneck, neuromorphic computing has emerged as a promising paradigm capable of highly parallel and energy‐efficient information processing for data‐intensive workloads [1, 2, 3, 4, 5]. Among the various hardware approaches, memristive devices based on two‐dimensional (2D) materials have attracted increasing attention for implementing artificial synapses. This interest stems from the intrinsic advantages of 2D materials, including atomically smooth surfaces, excellent mechanical flexibility, tunable electronic and optoelectronic properties, and strong device scalability, which collectively enable high‐performance and energy‐efficient neuromorphic devices [6, 7, 8]. Recent studies have demonstrated that 2D memristors can emulate key synaptic behaviors essential for neuromorphic computation. In particular, memristors exhibiting volatile and nonlinear dynamics are well suited for reservoir computing (RC), a computational framework derived from recurrent neural networks that efficiently processes temporal and sequential information while requiring training only at the readout layer [9, 10, 11].

Compared with conventional vertical 2D memristors employing a “sandwich” configuration, lateral 2D memristors offer greater design flexibility because the active channel lies within the plane of the material. This planar geometry enables more versatile device architectures, including multi‐terminal memtransistors and integrated optoelectronic functionalities, thereby expanding their applicability in neuromorphic and sensing systems [12, 13]. In general, lateral 2D memristors operate through two dominant switching mechanisms: interfacial barrier modulation and conductive filament formation. Among these, barrier‐modulation devices are particularly attractive because they rely on intrinsic material responses, such as ferroelectric polarization switching or electric‐field‐driven migration of internal defects, which enable analog switching behavior desirable for neuromorphic applications [14, 15, 16]. However, many reported devices require intentional defect engineering or pretreatment processes to activate memristive switching. For example, lateral memristive devices based on GeSe and ReS_2_ utilize vacancy‐mediated barrier modulation, where vacancies are artificially introduced through nitrogen‐ion irradiation or electron‐beam exposure [17, 18]. In addition, in‐plane anisotropic 2D materials, such as black phosphorus [19, 20], ReS_2_ [21, 22, 23], PdSe_2_ [24, 25, 26], and GaTe [27], offer opportunities to realize multifunctional and novel performances in lateral memristive devices owing to their direction‐dependent properties, such as polarization‐sensitive light absorption and anisotropic carrier transport [28, 29, 30, 31]. Such intrinsic anisotropy provides a new degree of freedom for engineering directionally programmable device functionalities. Indeed, pioneering studies have demonstrated orientation‐dependent synaptic sensing networks based on anisotropic materials such as Nb_2_GeTe_4_ and ReSe_2_, enabling biomimetic visual perception systems with polarization sensitivity [32, 33].

Recently, the layered niobium oxide dihalides NbOX_2_ (X = Cl, Br, and I) have attracted increasing interest as a new family of two‐dimensional van der Waals materials with pronounced in‐plane anisotropy. These materials exhibit intrinsic in‐plane ferroelectricity, originating from Peierls distortion along the Nb‐O‐Nb bonding direction, which gives rise to switchable polarization and rich anisotropic physical properties [34, 35]. In addition to their ferroelectric behavior, NbOX_2_ compounds display remarkable anisotropic optical responses, including layer‐independent second‐harmonic generation and near‐unity linear dichroism, highlighting their potential for polarization‐sensitive photonic and optoelectronic applications [36, 37]. Leveraging these unique properties, layered NbOCl_2_ has recently been demonstrated as a platform for spontaneous parametric down‐conversion quantum light sources, enabling highly compact on‐chip polarization‐manipulation components for integrated quantum photonics [38]. Despite these advances, most studies have focused on phenomena associated with the b‐axis, where the ferroelectric polarization is aligned. In contrast, the c‐axis, which represents the other principal in‐plane crystallographic direction, remains comparatively unexplored. As a result, the direction‐dependent electronic transport and device functionalities along the c‐axis of NbOX_2_ have yet to be systematically investigated, leaving a largely untapped opportunity for discovering new anisotropy‐enabled electronic and neuromorphic device behaviors.

In this work, we uncover intrinsic orientation‐dependent memristive switching in the in‐plane anisotropic van der Waals material NbOCl_2_ and demonstrate its potential for neuromorphic information processing. Remarkably, lateral NbOCl_2_ devices exhibit robust resistive switching exclusively along the crystallographic c‐axis without any channel pretreatment, achieving a current on/off ratio of ≈83 with stable cycle‐to‐cycle operation. Through a combination of systematic experimental characterizations and first‐principles calculations, we reveal that this anisotropic memristive behavior originates from direction‐dependent oxygen‐ion diffusion barriers coupled with ion‐migration‐induced modulation of the Schottky barrier at the metal‐semiconductor interface. Exploiting this intrinsic ion‐migration mechanism, the NbOCl_2_ memristor emulates key short‐term synaptic plasticity functions, including excitatory postsynaptic current (EPSC), paired‐pulse facilitation (PPF), spike‐amplitude‐dependent plasticity (SADP), and spike‐rate‐dependent plasticity (SRDP). Furthermore, the device's self‐relaxation and nonlinear dynamics are harnessed to implement a 4‐bit reservoir computing system, achieving handwritten digit recognition using the MNIST dataset. These results establish NbOCl_2_ as a promising anisotropic memristive platform and provide useful insights into other NbOX_2_ compounds, highlighting a new materials strategy for directionally programmable neuromorphic electronics.

## 2 Results and Discussion

### 2.1 Lattice Structure and Anisotropic Switching Behavior of NbOCl_2_

**FIGURE 1:**
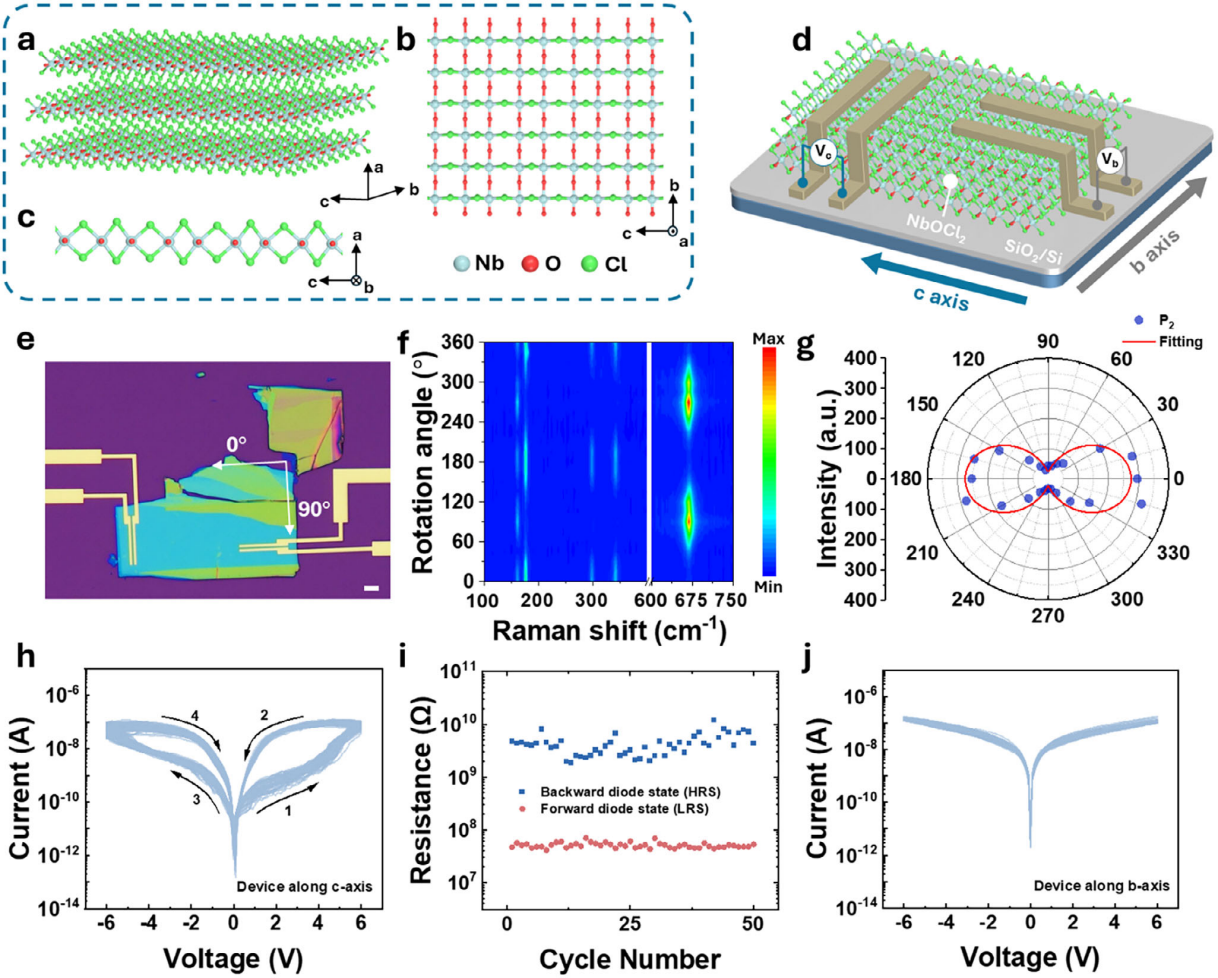
(a–c) Schematic of the crystal structure of 2D NbOCl_2_: (a) 3D view, (b) top view, and (c) side view. (d) Schematic and (e) Optical image of the NbOCl_2_ devices along the b‐axis and c‐axis directions. (f) Angle‐resolved Raman spectra for NbOCl_2_ in panel (e) in parallel polarized configuration. (g) Polar plot of parallel polarized Raman peak intensity (P_2_) as a function of the sample rotation angle. (h) *I*‐*V* curves of the NbOCl_2_ device along c‐axis during 50 voltage sweeps. The black arrows show the voltage sweep order. (i) Resistance values extracted from panel h) at 2 V. (j) *I*‐*V* curves of the NbOCl_2_ device along b‐axis during consecutive sweeps. Scale bar is 5 µm.

NbOCl_2_ crystallizes in a monoclinic layered structure belonging to the C2 space group, where the a‐axis is oriented out of plane, while the b‐ and c‐axes lie within the plane of the crystal lattice (Figure 1a). Each NbOCl_2_ monolayer is composed of a two‐dimensional network of Nb[O_2_Cl_4_] octahedra that share both corners and edges. Along the c‐axis, neighboring octahedras are interconnected through Cl‐Cl edge sharing, whereas along the b‐axis they are linked via oxygen bridges between adjacent Nb atoms (Figure 1b, c). This asymmetric bonding configuration gives rise to the intrinsic in‐plane structural anisotropy of NbOCl_2_. Due to the weak van der Waals (vdW) interactions between adjacent layers, thin NbOCl_2_ flakes can be readily exfoliated from bulk single crystals. The resulting flakes typically display a rectangular morphology dictated by the underlying crystal symmetry. Figure 1e presents an optical micrograph of two laterally configured two‐terminal devices fabricated on a SiO_2_ (285 nm)/Si substrate, each aligned along a different in‐plane crystallographic direction. Atomic force microscopy (AFM) measurements confirm that the NbOCl_2_ flake used in the devices has a thickness of approximately 35 nm (Figure S1). Both devices share identical channel dimensions, with a channel length of 500 nm and a width of 20 µm. Importantly, the device channels are aligned parallel to the edges of the rectangular flake, allowing one device to be oriented along the b‐axis and the other along the c‐axis. A three‐dimensional schematic illustrating the device configuration corresponding to the optical image in Figure 1e is shown in Figure 1d.

The in‐plane c‐ and b‐axes of the NbOCl_2_ flake in Figure 1e, with 0° and 90° defined as reference directions, were identified via angle‐resolved Raman spectroscopy in a parallel polarization configuration, as shown in Figure 1f. NbOCl_2_ exhibits five characteristic vibrational modes (P_1_‐P_5_) located at ≈160, ≈176, ≈297, ≈340, and ≈670 cm^−1^, respectively. Based on the angular‐dependent Raman intensity analysis (Figure 1g and Figure S2), modes P_2_, P_3_, P_4_, and P_5_ display two‐lobed polar patterns, whereas P_1_ exhibits a four‐lobed pattern. Notably, the intensities of P_2_, P_3_, and P_4_ reach maximum at 0° and 180°, while P_5_ shows minimum at these orientations. Following previous reports, 0° is defined as parallel to the c‐axis, whereas 90° corresponds to the b‐axis [38].

The device oriented along the c‐axis demonstrates stable forming‐free memristive switching characteristics over consecutive sweep cycles, as shown in Figure 1h, where the labels and arrows denote the sweep sequence and direction. As the voltage is swept from 0 to 6 V (stage 1), the device switches from the high‐resistance state (HRS) to the low‐resistance state (LRS), and the LRS is maintained as the voltage returns to 0 V (stage 2). The device resets to HRS after crossing the 0 V point and transitions to LRS during the sweep from 0 to ‐6 V (stage 3) and remains in the LRS as the voltage is brought back to 0 V (stage 4). In contrast, the *I*‐*V* curves of b‐axis device show no discernible hysteresis under the same ±6 V voltage sweeps (Figure 1j), confirming the anisotropic memristive behavior in NbOCl_2_. The Raman spectra collected from the channel region of the b‐axis device exhibit no discernible peak shifts following electrode fabrication (Figure S3), indicating that negligible strain is introduced during the process [38, 39]. Furthermore, prior studies on NbOI_2_—another member of the NbOX_2_ family—have shown that b‐axis devices display hysteresis‐free switching in the absence of strain, as strain‐free conditions result in weak spontaneous polarization. By analogy, the lack of hysteresis observed in the b‐axis NbOCl_2_ device can be attributed to similarly weak spontaneous polarization under strain‐free conditions [40].

The c‐axis device demonstrates average HRS and LRS resistance values of 4.35 × 10^9^ Ω and 5.1 × 10^7^ Ω at 2 V, respectively, corresponding to an on/off ratio of approximately 83 (Figure 1i). Notably, the on/off ratio depends on the sweep amplitude (Figure S4), with higher sweep voltages yielding larger on/off ratios. In addition, the backward‐to‐forward (B‐F) and forward‐to‐backward (F‐B) transition voltages were extracted from the nonlinearity of the *I*‐*V* characteristics, as detailed in Figure S5. The cycle‐to‐cycle variations analysis and statistical distribution of the B‐F and F‐B transition voltages are shown in Figure S6a, b, respectively, where the temporal variations are as low as 6.3% (B‐F) and 5.3% (F‐B), indicating good switching uniformity. Furthermore, the switching characteristics are largely independent of channel thickness, whereas the cycling stability shows a pronounced thickness dependence. As illustrated in Figure S7, devices with thinner channels exhibit noticeably degraded cycling stability.

### 2.2 Oxygen‐Ion‐Concentration‐Dependent Memristive Switching in NbOCl_2_

**FIGURE 2:**
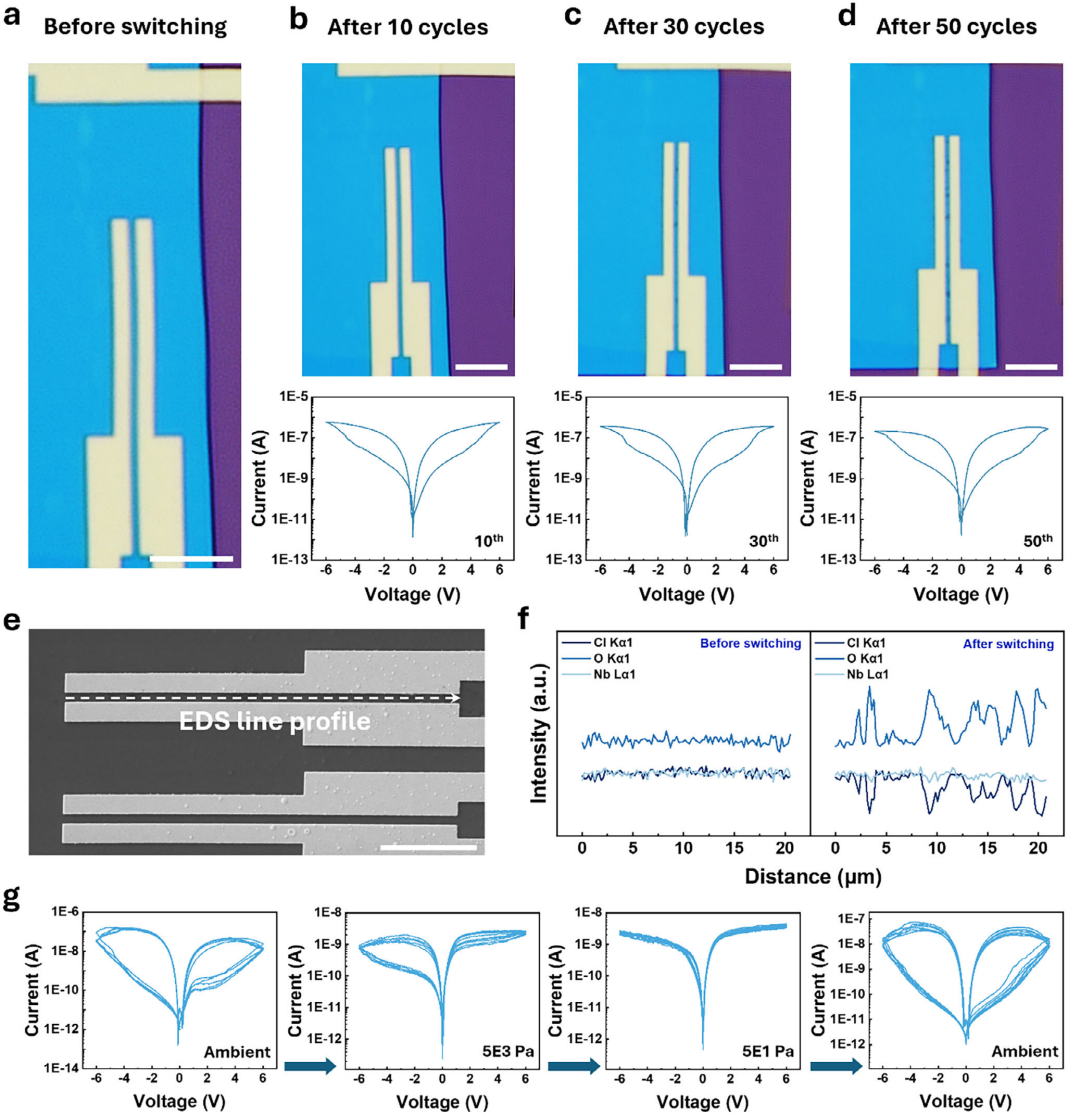
(a–d) Optical images and corresponding *I*‐*V* curve of the NbOCl_2_ device along c‐axis after different switching cycle processes: a) before switching, b) 10 cycles, (c) 30 cycles and (d) 50 cycles. (e) SEM image of NbOCl_2_ device along c‐axis. The white dashed line indicates the path of EDS line profile. (f) EDS line profile of Nb (light blue), O (blue), and Cl (dark blue) of the NbOCl_2_ device before and after switching. (g) *I*‐*V* curves of NbOCl_2_ lateral memristor under different atmospheric pressures. Scale bars are 5 µm.

To elucidate the switching mechanism of the lateral NbOCl_2_ memristor along the c‐axis, as well as the origin of its anisotropic memristive behavior, we monitored the structural and electrical evolution of the device during repeated voltage sweeps. The optical micrograph of the as‐fabricated c‐axis device is presented in Figure 2a, while the corresponding optical images and *I‐V* characteristics obtained after different numbers of switching cycles are shown in Figure 2b–d (The original optical microscopy images corresponding to Figure 2a–d are shown in Figure S8). As the number of voltage sweeps increases, the device maintains stable memristive switching behavior; however, several darkened regions progressively appear within the channel area. These regions become more pronounced with continued electrical stressing, indicating localized structural or compositional changes induced during the switching process. Notably, such darkened features are not observed in devices oriented along the b‐axis, even after dozens of voltage sweep cycles (Figure S9a, b). This clear orientation dependence strongly suggests that the formation of these darkened regions is closely correlated with the memristive switching mechanism along the c‐axis.

The Raman spectra acquired from the darkened area demonstrates reduced intensities in all characteristic peaks (P_1_‐P_5_) after voltage sweeps, as shown in Figure S10a–d, indicating a local phase transition from a crystalline to an amorphous state within the darkened regions. In addition, previous studies have suggested that such reduction of Raman intensities is often associated with material oxidation [41, 42]. The darkened regions observed in the optical micrograph can also be clearly distinguished in the scanning electron microscope (SEM) image (Figure S11a). Furthermore, energy‐dispersive X‐ray spectroscopy (EDS) analysis of the whole channel region before and after device switching probes the compositional changes of Cl, O, and Nb in darkened areas as shown in Figure 2f. Importantly, Si_3_N_4_ substrates were employed in EDS analysis instead of SiO_2_, facilitating more reliable differentiation of oxygen‐related signal variations. Even with this approach adopted, the initial O signal is still higher than the other two elements due to the presence of oxygen in the Si_3_N_4_ substrate. The elemental weight‐intensity line profile is collected along the dashed arrow in Figure 2e, which reveals an increased O concentration accompanied by a reduced Cl concentration within the darkened regions. This compositional fluctuation in the line profile agrees well with the EDS mapping results (Figure S11b–d), confirming the occurrence of oxidation in these darkened regions.

In addition, the transport characteristics of the device along the c‐axis were measured under different atmospheric pressures (Figure 2g). The memory window gradually decreases with decreasing pressure and recovers once the device is returned to ambient conditions. This pressure‐dependent switching is consistent with the prior report on oxide‐based memristors, in which oxygen ion migration becomes inefficient under vacuum and therefore cannot be effectively driven by an external electric field [43]. Furthermore, the Kelvin probe force microscopy (KPFM) topography image and surface potential image demonstrate the work function difference between Pd and NbOCl_2_ with a 5 nm Pd layer deposited on the NbOCl_2_ flake (Figure S12a, b). The line profile in Figure S12c shows that the Pd/NbOCl_2_ region has a work function 0.382 eV lower than that of pristine NbOCl_2_, indicating the formation of a Schottky barrier at the device contact. Thus, we deduce that the darkened regions provide diffusion pathways for oxygen‐ion migration under an external electric field, and that the switching behavior arises from oxygen‐ion‐migration‐induced modulation of the Schottky barrier height (SBH). Notably, the decrease in Raman peak intensity becomes much less pronounced upon further cycling (500 cycles), accompanied by a reduced switching on/off ratio (Figure S10). This evolution suggests a gradual saturation of bulk oxidation, which weakens the effective modulation of the interfacial barrier.

### 2.3 Theoretical Calculations of the Anisotropic Memristive Behaviors in NbOCl_2_

**FIGURE 3:**
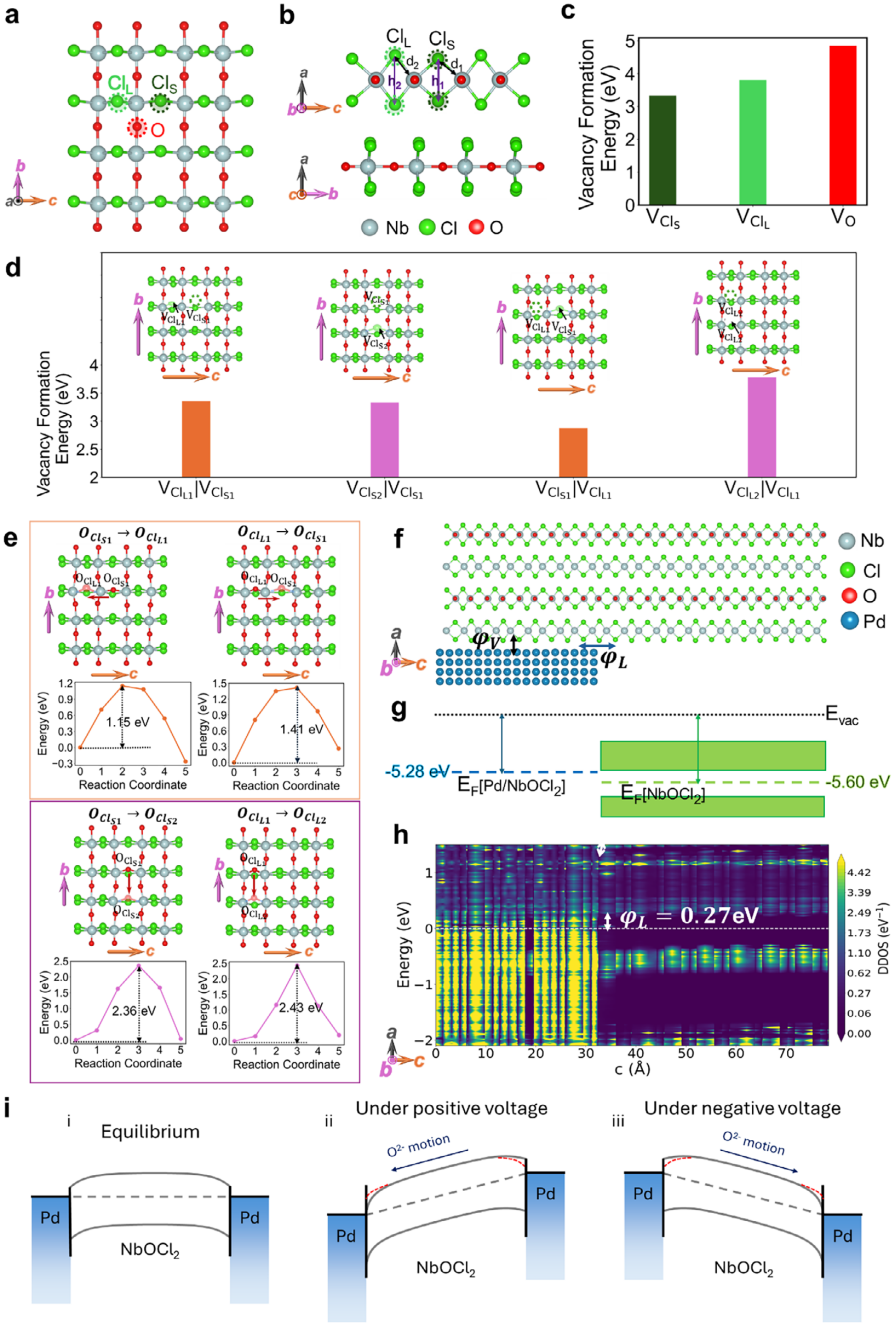
(a) Top‐view and (b) Side‐view images of the atomic structure of the NbOCl_2_ monolayer. Cl atoms are categorized into two distinct sites: Cl_S_ and Cl_L_. (c) Calculated formation energies for isolated vacancies at Cl_S_, Cl_L_, and O sites. V_X_ denotes the vacancy at the site X. (d) Bar plot illustrating the conditional formation energy for a secondary Cl vacancy. The notation V_ClX_|V_ClY_ denotes the energy required to create a vacancy at site X given that a vacancy already exists at site Y. (e) Energy barrier profiles derived from Nudged Elastic Band (NEB) calculations for the migration of an adsorbed O atom between neighboring Cl vacancies. O_ClX_ denotes an O atom adsorbed at a Cl vacancy site X. (f) Atomic snapshot of Pd (4‐layer)‐NbOCl_2_ (4‐layer) device configuration, which shows both vertical and lateral interface between Pd and NbOCl_2_. *φ*_V_ and *φ*_L_ denote the vertical and lateral SBHs, respectively. (g) Fermi‐level alignment of the Pd/NbOCl_2_ heterostructure and pristine NbOCl_2_. The blue and green dashed lines indicate the Fermi levels of Pd/NbOCl_2_ and NbOCl_2_, respectively. (h) Local density of states across the Pd‐NbOCl_2_ device calculated via quantum transport simulations. (i) Schematic band diagrams illustrating the three different states during device switching. The red dash lines indicate the variation of barrier height.

Here, we employed density functional theory (DFT) calculations to further elucidate the microscopic origin of the planar dependence of hysteresis observed in the device. Experimental data indicate an increase in O concentration and a decrease in Cl concentration during the transition to the low‐resistance state, strongly suggesting that O atoms adsorb at Cl vacancy sites. However, to rationalize the directional dependence of the hysteresis, it is necessary to understand how oxygen adsorption and migration differ along different crystallographic orientations. We first analysed the atomic structure of the NbOCl_2_ monolayer and identified the most probable native vacancy sites. The NbOCl_2_ lattice contains two non‐equivalent chlorine environments, denoted as Cl_S_ and Cl_L_ in both top‐view and side‐view (Figure 3a,b). The Nb‐Cl_S_ and Nb‐Cl_L_ bond lengths are 2.53 and 2.45 Å respectively, while the vertical separations between the upper and lower Cl planes are 3.34 Å (h_1_) for the Cl_S_ sites and 3.88 Å (h_2_) for the Cl_L_ sites (Figure 3b). These structural differences indicate two distinct Cl coordination environments and contribute to the intrinsic anisotropy of the material. Along the c‐axis direction, the lattice is composed of alternating Cl_S_ and Cl_L_ atoms in a sequential arrangement. Whereas, along the b‐axis direction, the atomic rows consist of uniform Cl types.

Structural relaxations revealed that Nb vacancies induce severe local distortions, making their formation energetically and structurally unfavourable. Among the remaining defects, the Cl_S_ vacancy exhibits the lowest formation energy, indicating that native vacancies are most likely to occur at Cl_S_ sites (Figure 3c). The formation energy of a Cl_L_ vacancy is ≈0.5 eV higher than that of a Cl_S_ vacancy. This difference originates from the stronger Nb‐Cl_L_ bonding, as reflected by a larger integrated crystal orbital Hamilton population (ICOHP) value of 1.66 compared to 1.45 for Nb‐Cl_S_. These results suggest that native vacancies are predominantly located at Cl_S_ positions, providing favourable adsorption sites for oxygen. Upon the application of voltage, O atoms from the ambient environment can be adsorbed into the preexisting Cl vacancy sites. In addition, the applied bias may promote the formation of secondary vacancies, enabling further oxygen adsorption.

As the hysteresis is only observed along the c‐axis direction, we investigated both vacancy propagation tendencies and oxygen migration barriers to clarify the origin of this orientation dependence. The conditional vacancy formation energies, defined as the energies required to form a second Cl vacancy in the presence of a neighbouring vacancy, were calculated (Figure 3d). For clarity, we labelled the subsequent Cl sites in the b‐axis direction as Cl_S1_, Cl_S2_ and Cl_L1_, Cl_L2_. When a vacancy is present at Cl_S1_, the formation energies for generating an additional vacancy at Cl_L1_ (along c‐axis) and Cl_S2_ (along b‐axis) are nearly identical. However, once a vacancy forms at Cl_L1_, the formation energy for creating another vacancy at Cl_L2_ (along b‐axis) becomes significantly higher than that for forming a vacancy at Cl_S1_ (along c‐axis). This energy disparity indicates that vacancy propagation is strongly favoured along the c‐axis direction, enabling the formation of extended vacancy chains under bias. In contrast, vacancy generation along the b‐axis direction is energetically suppressed, limiting the development of continuous vacancy pathways required for resistive switching.

Since hysteresis requires not only vacancy availability but also efficient oxygen redistribution, oxygen migration barriers between neighbouring vacancy sites were calculated using climbing‐image nudged elastic band (CINEB) calculations (Figure 3e). The migration barriers for an O atom hopping between Cl_S1_ and Cl_L1_ vacancy sites along the c‐axis are lower, allowing oxygen to move readily in response to the applied switching voltage. In contrast, along the b‐axis, migration barriers between equivalent vacancy sites (Cl_S1_→Cl_S2_ or Cl_L1_→Cl_L2_) are substantially higher, effectively blocking oxygen redistribution along the b‐axis. Collectively, the combined preference for vacancy chain formation and low‐barrier oxygen migration along the c‐direction, which is determined by the intrinsic structural anisotropy of NbOCl_2_, provides a microscopic explanation for the experimentally observed direction‐dependent characteristics.

In the present device configuration, the Pd electrodes establish both vertical and lateral contacts with the NbOCl_2_ channel (Figure 3f), giving rise to two potential interfacial barriers: one associated with the vertical metal‐semiconductor interface and the other with the lateral contact region along the transport direction. To distinguish their respective roles, the vertical interface was first analysed through density functional theory (DFT) band‐structure calculations (Figure S13). The calculated band alignment indicates that the conduction band of NbOCl_2_ intersects the Fermi level at the vertical Pd/NbOCl_2_ interface, suggesting the formation of an Ohmic‐like contact in the out‐of‐plane direction. To assess the origin of the experimentally observed Schottky behavior, we calculated the work functions of the Pd/NbOCl_2_ heterostructure and pristine NbOCl_2_ (Figure 3g). The Pd/NbOCl_2_ heterostructure exhibits a work function of 5.28 eV, whereas pristine NbOCl_2_ shows a higher value of 5.60 eV, giving a work‐function difference of 0.32 eV, which is consistent with KPFM analysis. Although the Schottky‐Mott rule suggests that the conduction band minimum of NbOCl_2_ should lie very close to the Fermi level of the Pd/NbOCl_2_ heterostructure (Figure 3g), implying a negligibly small lateral Schottky barrier, such an approximation is often unreliable. In realistic metal/semiconductor interfaces, interfacial charge transfer and dipole formation can significantly modify the band alignment and barrier height. Indeed, the vertical Pd/NbOCl_2_ interface already exhibits substantial charge redistribution (Figure S13a), indicating that similar effects may also arise at the lateral junction. Therefore, an explicit first‐principles evaluation of the lateral contact is required. However, conventional periodic DFT calculations are not suitable for accurately capturing the electronic structure of a lateral device interface.

Thus, we performed quantum transport simulations with the lateral Pd‐NbOCl_2_ device model within the DFT+NEGF (non‐equilibrium Green's function) framework to calculate the device density of states along the c‐axis direction (Figure 3h). The calculated local density of states shows that the device Fermi level shifts toward the conduction band of NbOCl_2_, indicating that the Pd/NbOCl_2_ region possesses a lower work function than pristine NbOCl_2_, consistent with the experimental observation. However, in contrast to the Schottky‐Mott prediction, the conduction band minimum of NbOCl_2_ remains above the Fermi level with a finite energy separation, confirming the existence of a Schottky barrier at the lateral interface. By extracting the energy offset between the conduction band minimum and the Fermi level, we estimate a lateral SBH of approximately 0.27 eV. Overall, these simulation results confirm that the anisotropic memristive behavior in NbOCl_2_ lateral device originates from direction‐dependent energy barriers of oxygen‐ion diffusion and ion‐migration‐related interfacial barrier modulation.

To systematically illustrate the switching mechanism of the lateral NbOCl_2_ memristor, the hysteretic *I*‐*V* characteristics in the linear‐current regime was analyzed, as shown in Figure S14a, where the black dashed line denotes the full current loop, whereas the blue and red traces correspond to the forward‐diode (FD) and backward‐diode (BD) states, respectively. For clarity, the initial state of the lateral NbOCl_2_ memristor can be modeled as two back‐to‐back diodes in series with a constant resistance, as depicted in Figure S14b(i), where a bias is applied to the left electrode and the right electrode is grounded. Under a positive bias, the electric field drives substantial drift of negatively charged oxygen ions from the right electrode toward the left electrode. The resulting accumulation of oxygen ions reduces the work function of NbOCl_2_, thereby increasing (decreasing) the SBH at the left (right) contact. Conversely, under a negative bias, oxygen ions drift toward the right electrode, leading to an increased (decreased) SBH at the right (left) contact. Such switching behaviors can also be described using two alternative equivalent‐circuit models composed of asymmetric back‐to‐back diodes (Figure S14b(ii, iii)), associated with the FD and BD states, respectively. Furthermore, the band diagrams corresponding to all these three equivalent‐circuit models are depicted in Figure 3i. The proposed mechanism may also be applicable to other NbOX_2_ compounds, including NbOBr_2_ and NbOI_2_, which possess similarly anisotropic crystal structures [34, 35]. As such, these materials may exhibit comparable anisotropic memristive characteristics. Nevertheless, the detailed switching behavior is expected to vary with the halide composition, warranting further systematic investigation in future studies.

### 2.4 Short‐Term Memory Characteristics of NbOCl_2_ memristors in Transient Measurement

**FIGURE 4:**
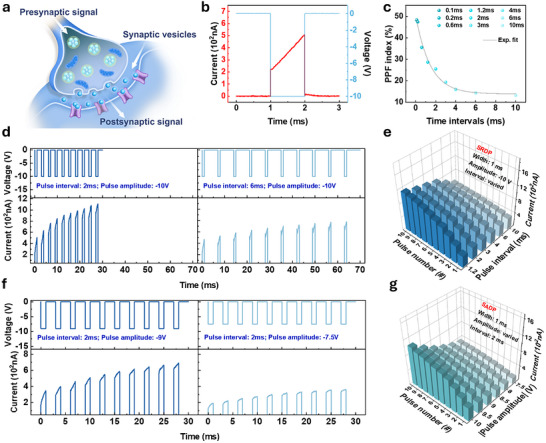
(a) Schematic illustration of a biological synapse. (b) Synaptic EPSC functions triggered by single spike. (c) PPF index analysis. (d) Dependence of current responses observed under different pulse intervals. (e) SRDP behavior triggered under pulse sequences with an amplitude of ‐10 V. (f) Dependence of current responses observed under different pulse amplitudes. (g) SADP behavior triggered under pulse sequences with interval of 2 ms.

In biological neural systems, synapses act as the fundamental interfaces for information transmission between neurons, where signals are communicated through the release of neurotransmitters from synaptic vesicles (Figure 4a). The strength of signal transmission, commonly referred to as the synaptic weight, can be dynamically modulated by the magnitude and frequency of external stimuli, which constitutes a key manifestation of synaptic plasticity. Analogously, in the lateral NbOCl_2_ memristor, the application of an external voltage drives the migration of defects, thereby modulating the SBH at the NbOCl_2_/electrode interfaces and enabling the emulation of synaptic functionalities. In this configuration, the left electrode functions as the presynaptic terminal, while the right electrode is grounded, and the measured channel current is defined as the EPSC. Figure 4b presents the EPSC response of the device triggered by a single voltage pulse (‐10 V, 1 ms), corresponding to an energy consumption of approximately 3.56 nJ, which is comparatively low relative to previously reported memristive synaptic devices (Table S1). Following the stimulation pulse, the EPSC gradually decays over several milliseconds under a read voltage of ‐1 V, indicating a transient conductance relaxation process (Figure S15). This behavior can be attributed to the gradient‐driven back‐diffusion of oxygen ions after removal of the external electrical pulse stimulus [44], and it closely resembles the short‐term plasticity (STP) observed in biological synapses.

Furthermore, as PPF is a hallmark of biological STP, we employed PPF to evaluate the temporal enhancement of synaptic weight in our device, mimicking the time‐dependent coupling of spike pairs in biological synapses. Two successive pulses (‐10 V, 1 ms) were applied with varying inter‐pulse intervals. The responses for intervals of 0.1, 0.2, and 0.6 ms are shown in Figure S16a, those for 1.2, 2, and 3 ms in Figure S16b, and those for 4, 6, and 10 ms in Figure S16c. The PPF index was obtained based on Equation (1) [45],

(1) $$PPF\;index\;\left(\%\right)=\frac{I_{2}-I_{1}}{I_{1}}\;\times 100$$

where *I*_1_ denotes the current response to the first pulse and *I*_2_ denotes that to the second pulse. The obtained PPF index decreases with increasing inter‐pulse interval, as shown in Figure 4c, indicating diminished facilitation at longer delays between pulses, and the data were fitted using a single‐exponential decay function (Equation (2)) [45],

(2) $$y=A+Bexp\;\left(\frac{-\Delta t}{\tau }\right)$$

where *A* represents the baseline conductance, *B* the amplitude of the exponential component, *Δt* the time elapsed after stimulation, and *τ* the relaxation time constant. The excellent fit suggests that relaxation is primarily governed by a single first‐order process, consistent with the redistribution of mobile oxygen ions at the NbOCl_2_/Pd interface, confirming the capability of the device to emulate STP.

Moreover, the device response to pulse trains, consisting of ten consecutive pulses (‐10 V, 1 ms) with inter‐pulse intervals of 2 ms and 6 ms, was investigated, as shown in Figure 4d. A more pronounced EPSC enhancement is observed at the shorter interval, consistent with the PPF results. Thus, the PPF behavior can be further extended to SRDP, as illustrated in Figure 4e. Pulse trains with inter‐pulse intervals ranging from 1.2 to 10 ms were applied to the lateral NbOCl_2_ memristor, yielding progressively larger current responses at higher spike rates. Such SRDP characteristics closely resemble the rate‐dependent synaptic responses observed in biological systems. Similarly, SADP was evaluated to further assess the synaptic emulation capability of the device (Figure 4f,g). Pulse trains comprising ten identical 1 ms stimulation pulses with amplitudes varying from ‐7.5 V to ‐10 V were applied, and a markedly larger current response is obtained at higher pulse amplitudes. These predictable SRDP and SADP behaviors are desirable features for enabling reliable neuromorphic applications.

### 2.5 MNIST Handwritten Digit Recognition With a 4‐Bit RC Framework

**FIGURE 5:**
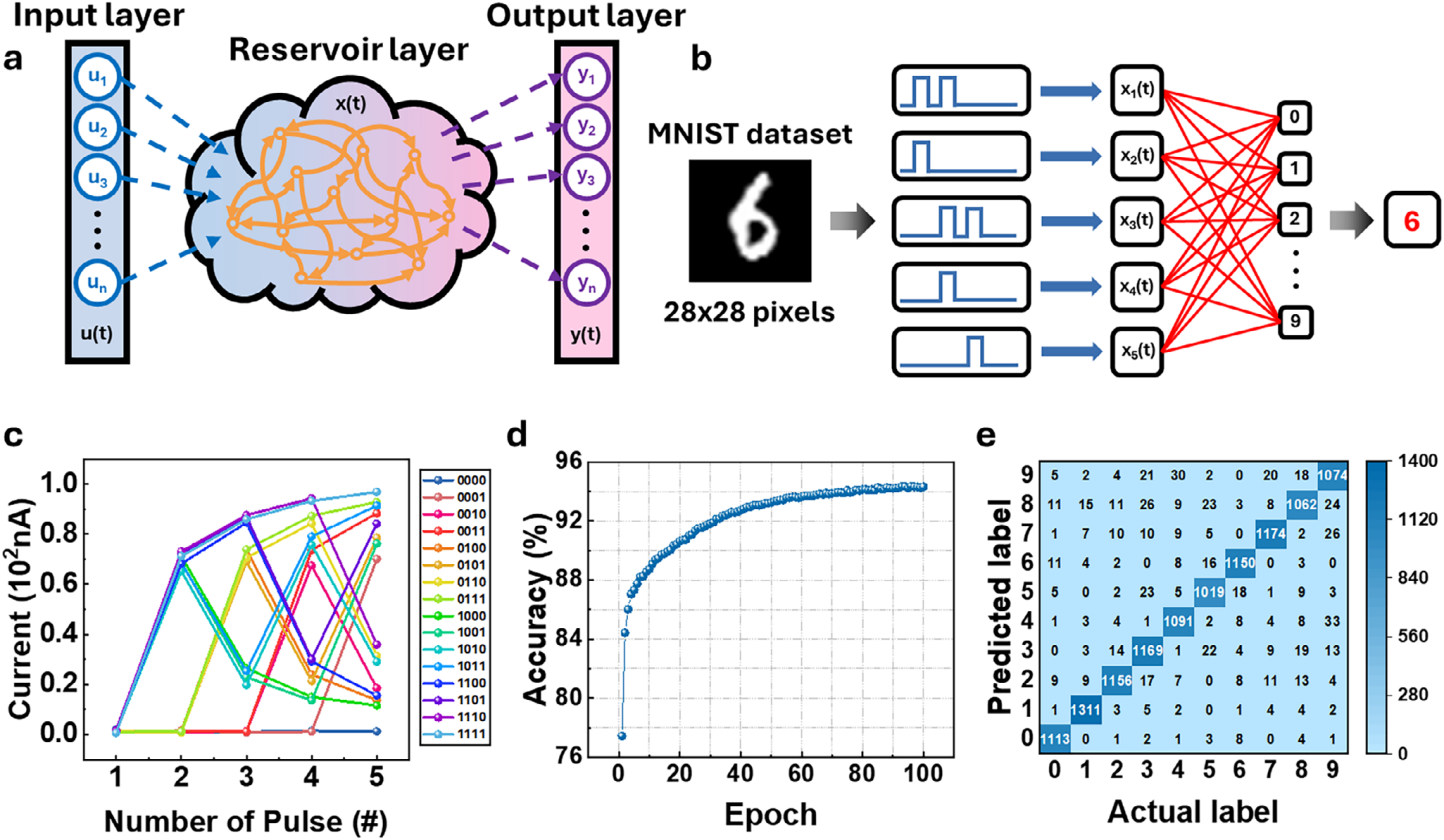
(a) Schematic illustration of RC. (b) Memristor‐based RC using the application of pulse schemes to the memristor, enabling letter representation. (c) 4‐bit RC achieved using the NbOCl_2_ memristor. (d) Accuracy of pattern recognition achieved using the RC system. (e) Confusion matrix generated from the simulation results.

To evaluate the potential of the lateral NbOCl_2_ memristor for neuromorphic computing, we leveraged the short‐term memory dynamics of the device within a RC framework. RC is a machine‐learning paradigm particularly effective for processing time‐dependent and sequential signals, and it typically comprises three functional components: the input layer, the reservoir, and the readout layer [46, 47], as illustrated in Figure 5a. In this architecture, a nonlinear dynamical reservoir possessing intrinsic short‐term memory transforms temporal input signals into a high‐dimensional state space, enabling efficient representation of temporal features. Importantly, only the readout layer, which maps the reservoir states to the desired outputs, requires training using conventional learning algorithms, thereby significantly simplifying the training process [9, 46]. Here, the lateral NbOCl_2_ memristor serves as the physical reservoir, exploiting its self‐relaxation and nonlinear conductance dynamics to encode temporal information. The device was integrated into an RC system to perform pattern‐recognition tasks based on the MNIST dataset, with the overall classification workflow illustrated in Figure 5b.

Designed pulse trains comprising four pulses were applied to the device to implement 4‐bit RC with 16 reservoir states (Figure 5c). Binary inputs “1” and “0” were encoded as ‐10 V and 0 V pulses, respectively, and the device states were read out with a ‐1 V read pulse. In addition, the initial current level for each of the 16 states was also obtained, enhancing the reliability of state encoding. The input images were first binarized according to the grayscale value of each pixel, encoded into 4‐bit pulse sequences, and then applied to the reservoir layer, followed by classification via the readout layer. The recognition accuracy reached 94.3% after 100 epochs (Figure 5d), and the corresponding confusion matrix was obtained after training and testing, as shown in Figure 5e, providing a class‐by‐class comparison between the true labels and the predicted outputs across all 10 categories. Collectively, these results highlight the strong potential of the lateral NbOCl_2_ memristor for neuromorphic information processing.

## 3 Conclusions

In summary, we have demonstrated anisotropic memristive switching in the emerging 2D van der Waals material NbOCl_2_ and its application in neuromorphic information processing. Lateral devices exhibit pronounced resistive switching along the in‐plane c‐axis, achieving a stable current on/off ratio with reproducible cycling, whereas no memristive behavior is observed along the b‐axis, revealing a strong crystallographic direction dependence. Combined experimental characterization and first‐principles analysis identify the origin of this anisotropic switching as oxidation‐assisted, electric‐field‐driven oxygen‐ion migration, which modulates the Schottky barrier at the NbOCl_2_/electrode interface through direction‐dependent ion diffusion pathways. Leveraging this dynamic ion‐migration mechanism, the NbOCl_2_ memristor exhibits self‐relaxation conductance dynamics capable of emulating key short‐term synaptic plasticity behaviors. Furthermore, a 4‐bit reservoir computing system based on the device achieves 94.3% accuracy in handwritten digit recognition. These results establish anisotropic ion migration in NbOCl_2_ as a new route toward directionally programmable memristors, offering a promising materials platform for next‐generation neuromorphic and in‐memory computing systems.

## 4 Experimental Section

### 4.1 Sample Preparation and Device Fabrication

The NbOCl_2_ thin flakes were mechanically exfoliated from the bulk single crystal and then transferred onto the SiO_2_ (285 nm)/Si substrates using a polydimethylsiloxane (PDMS)‐assisted dry‐transfer method. Then, an electron‐beam lithography process was used to form electrode patterns, and a Pd/Au (25/30 nm) thin film was deposited by the electron beam evaporation process to form the electrodes. Finally, remover PG and isopropanol were used in lift‐off process to remove the PMMA resist film, completing the fabrication of the lateral NbOCl_2_ two‐terminal devices.

### 4.2 Characterizations and Measurements

The morphology and thickness measurements were performed in an AFM system (Bruker, Dimension Icon) with tapping mode. KPFM measurements were conducted using the same AFM system in PeakForce KPFM mode. Polarization‐dependent Raman measurements were conducted by rotating the sample with a rotation stage while maintaining the polarizer in fixed configuration. Raman signals were collected with a 100× objective and an exposure time of 10 s, using a 532 nm laser scource. SEM and EDS analyzes were performed using Hitachi Regulus 8230 at an acceleration voltage of 5 kV. The electrical characterizations, including DC *I*‐*V* and pulse *I*‐*V* measurements, were done by the Keysight B1500A semiconductor analyzer under the dark ambient environment at room temperature. A turbo probe station containing vacuum gauge was utilized for DC measurement under vacuum. The designed voltage waveforms for pulse *I*‐*V* measurements was generated by the Keysight B1530, with a waveform generator fast measurement unit (WGFMU) installed in the analyzer.

### 4.3 Theoretical Calculations

DFT calculations were performed using the Vienna ab initio simulation package (VASP) [48] with the projector augmented‐wave (PAW) [49] method and the GGA‐PBE [50] exchange correlation functional. A 2×4 supercell of NbOCl_2_ was built along the in‐plane c and b directions, yielding lattice parameters of ≈13.5 and ≈15.7 Å, respectively. A plane‐wave cutoff energy of 520 eV was used, and all calculations were spin‐polarized. Structural relaxations were performed using the Quick‐Min optimizer [51] with convergence criteria of 10^−5^ eV for electronic self‐consistency and 0.04 eV/Å for ionic forces. Γ‐point sampling was used during relaxations, while a 2 × 2 × 1 K‐point mesh was adopted for single‐point energy calculations. Dipole correction was applied along the out‐of‐plane direction. The formation energy for elemental defects was calculated using the following equation, 𝐸_𝑓_ = 𝐸_𝑣_ − 𝐸_𝑠𝑢𝑝𝑒𝑟_ + 𝜇_𝑖_, where 𝐸_𝑣_ and 𝐸_𝑠𝑢𝑝𝑒𝑟_ were the total energies of the defected and pristine NbOCl_2_ supercell, respectively; 𝜇_𝑖_ was the chemical potential of the vacancy element. The chemical potentials of Cl and O atoms were obtained from half the total energies of Cl_2_ and O_2_ molecules, respectively. Migration barriers were calculated using the CI‐NEB [52] method implemented in VTST tools, with a spring constant of 5 eV/Å^2^ and the same convergence parameters as used for relaxation. The COHP analysis has been performed using LOBSTER [53] package.

For vertical Pd/NbOCl_2_ contact analysis, a heterostructure consisting of four layers of Pd(100) and four layers of NbOCl_2_ was constructed using the QuantumATK interface builder [54]. A 5×2 Pd supercell was rotated by 45° to match a 3×1 NbOCl_2_ supercell along the c‐ and b‐directions, respectively, resulting in a mean strain of 0.76% (Figure S13). The vdW interactions between the layers were included using the DFT‐D3 method of Grimme with Becke‐Johnson damping. A vacuum spacing of 20 Å was added along the out‐of‐plane direction. Band structures and work functions were extracted using py4vasp and VASPKIT [55], and atomic structures were visualized using VESTA.

Quantum transport simulations for the lateral Pd/NbOCl_2_ contact were performed using QuantumATK. The device was constructed by extending the Pd/NbOCl_2_ heterostructure along the c‐direction to a total length of ≈78.5 Å, with the Pd‐covered region (≈31.5 Å) connected to a pristine NbOCl_2_ region (≈47 Å) (Figure 3f). The left and right electrodes consisted of Pd/NbOCl_2_ and pristine NbOCl_2_, respectively, each with a length of ≈19.6 Å. A double‐zeta polarized (DZP) basis set was used with a cutoff energy of 60 Ha. Dirichlet boundary conditions were applied along the transport direction (c‐direction), with 100 k‐points along direction c, 5 along periodic direction b, and 1 along out‐of‐plane direction a.

### 4.4 Dataset and Readout Function model

The MNIST dataset contains 60,000 images (28 × 28 pixels). We used 80% of the dataset for training and the remaining 20% for testing. The readout layer was trained using softmax regression (SR) implemented with the Scikit‐learn package in Python.

## Author Contributions

**Yufei Shi**: data analysis. **Sanchali Mitra**: device simulation and analysis. **Caokun Wang**: device fabrication and measurement, data analysis, writing – original draft. **Yun Ji**: data analysis, writing – review and editing. **Haofei Zheng**: device fabrication. **Kah‐Wee Ang**: supervision, writing – review and editing, funding acquisition. **Yee Sin Ang**: supervision, writing – review and editing.

## Conflicts of Interest

The authors declare no conflict of interest.

## Supporting information




**Supporting File**: advs76461‐sup‐0001‐SuppMat.pdf.

## Data Availability

The data that support the findings of this study are available from the corresponding author upon reasonable request.
