# Supplementary material for: Anisotropic Memristive Switching in NbOCl2 Enabled by Directional Oxygen Ion Migration
Source: Adv Sci (Weinh). 2026 Jul 13:e76461. Online ahead of print. doi: 10.1002/advs.76461 (PMC13360125; doi:10.1002/advs.76461)
Supplement: Supplementary file 1 — Supporting File: advs76461‐sup‐0001‐SuppMat.pdf. [file ADVS-9999-e76461-s001.pdf]

Supporting Information

Anisotropic Memristive Switching in $NbOCl_2$ Enabled by Directional Oxygen Ion Migration

Caokun Wang, Yun Ji, Sanchali Mitra, Yufei Shi, Haofei Zheng, Yee Sin Ang and Kah-Wee Ang**

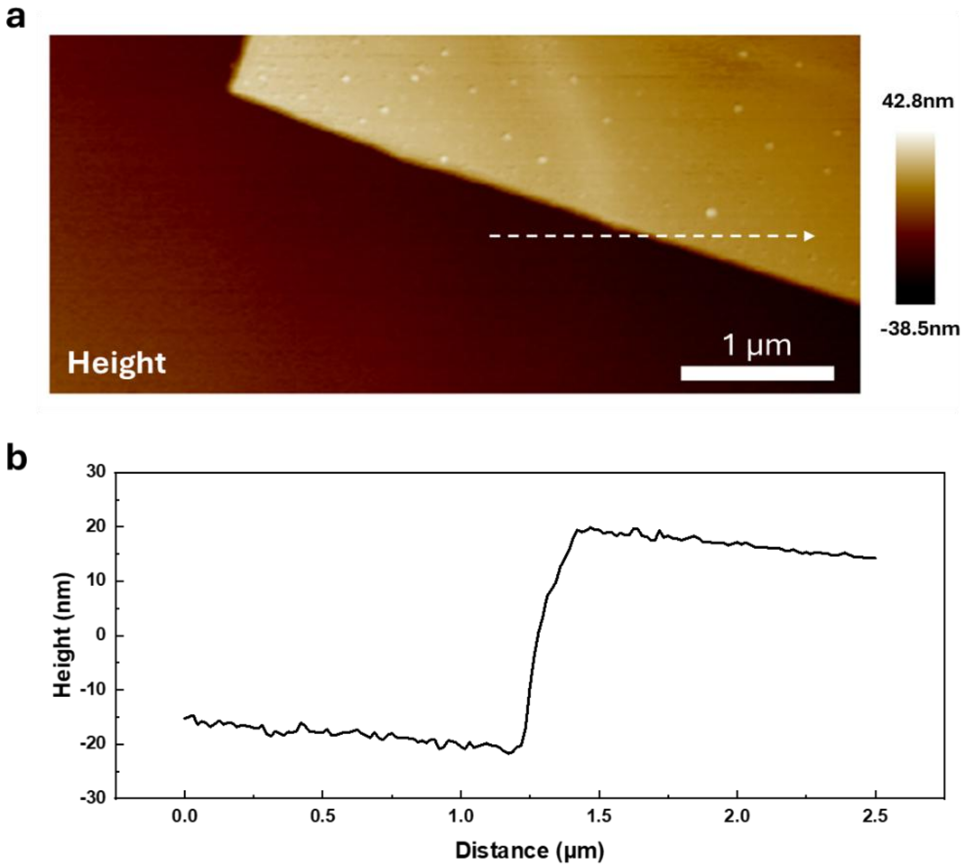

Figure S1. a) AFM topography image of the $NbOCl_2$ flake on SiO_2/Si substrate. White dash arrow indicates the path of AFM line profile. b) AFM line profile data along the white dash arrow in panel a).

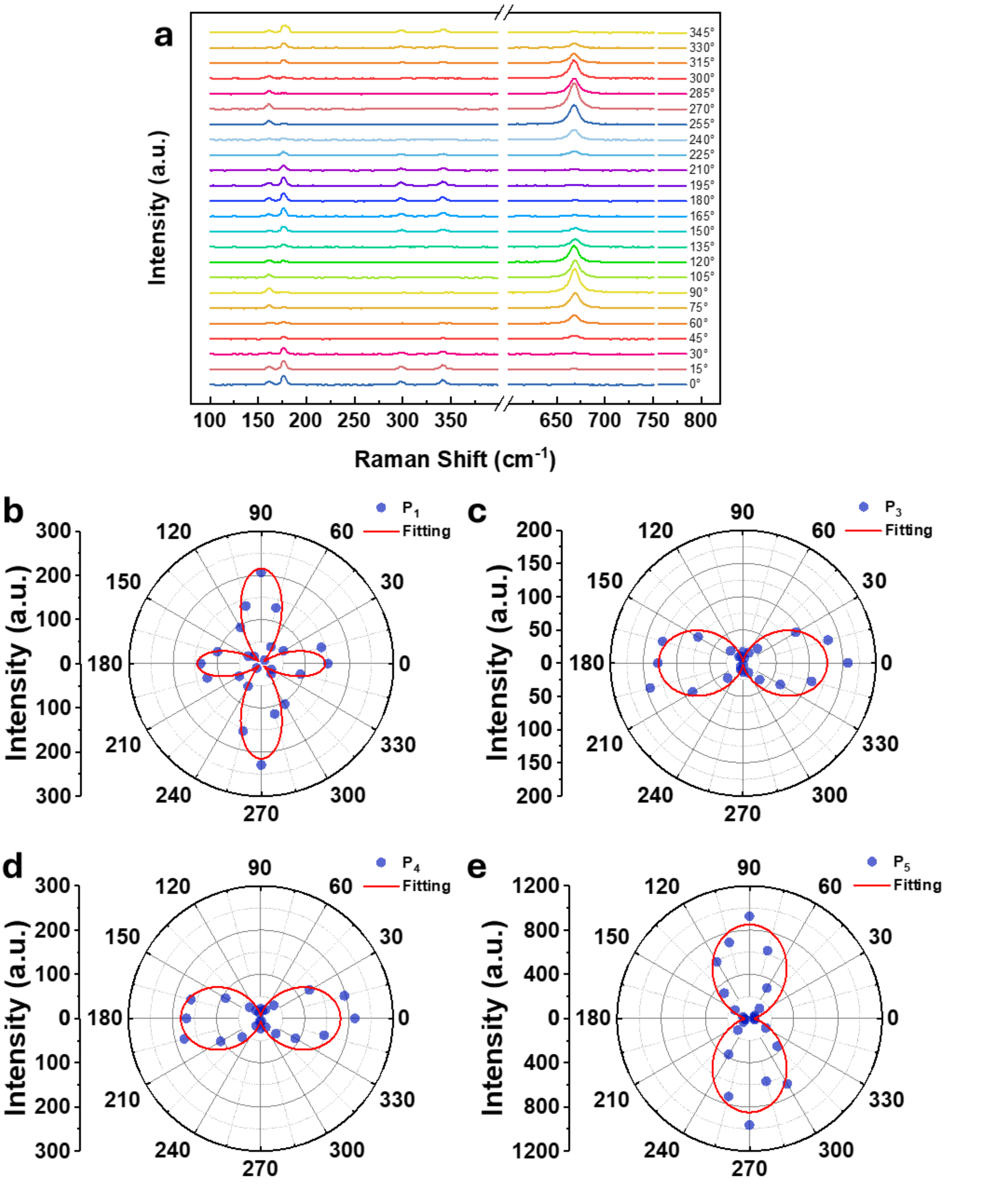

Figure S2. Angle-resolved Raman Spectra. a) Raman spectra of the $NbOCl_2$ flake in Figure 1e at various polarization angles under parallel configuration. b-e) Polarization-dependent Raman peak intensity of P_1, P_3, P_4 and P_5, respectively.

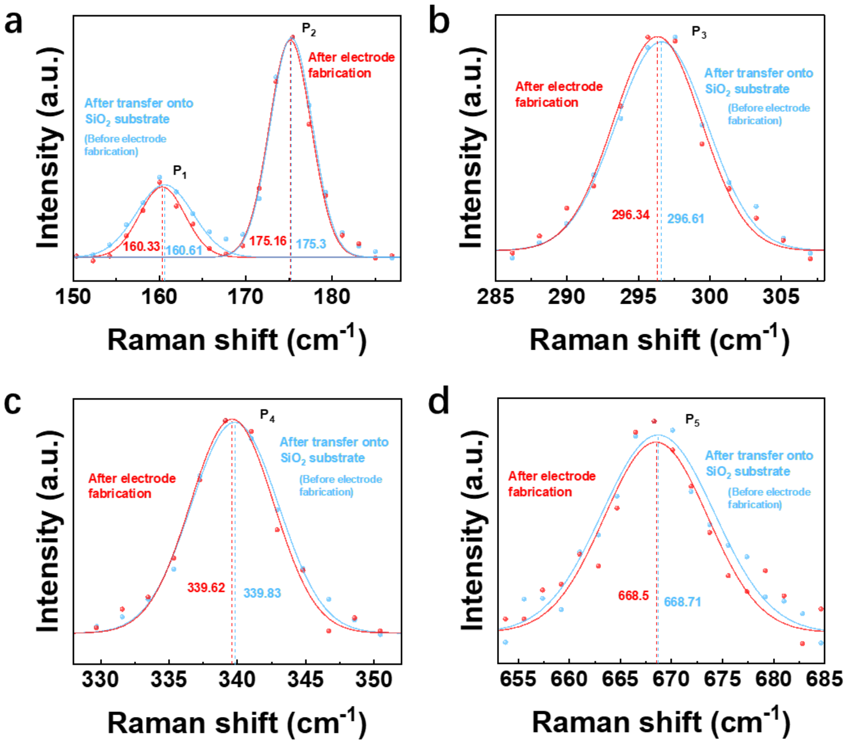

Figure S3. Normalized Raman spectra of different peaks of $NbOCl_2$ before (blue curve) and after (red curve) electrodes fabrication: a) P_1 and P_2, b) P_3, c) P_4 and d) P_5.

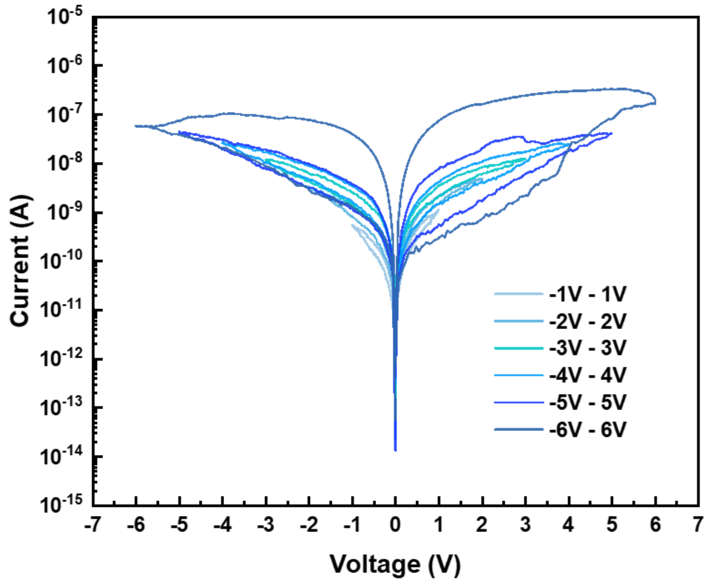

Figure S4. *I-V* characteristics of $NbOCl_2$ lateral memristor along c-axis under different voltages sweeping.

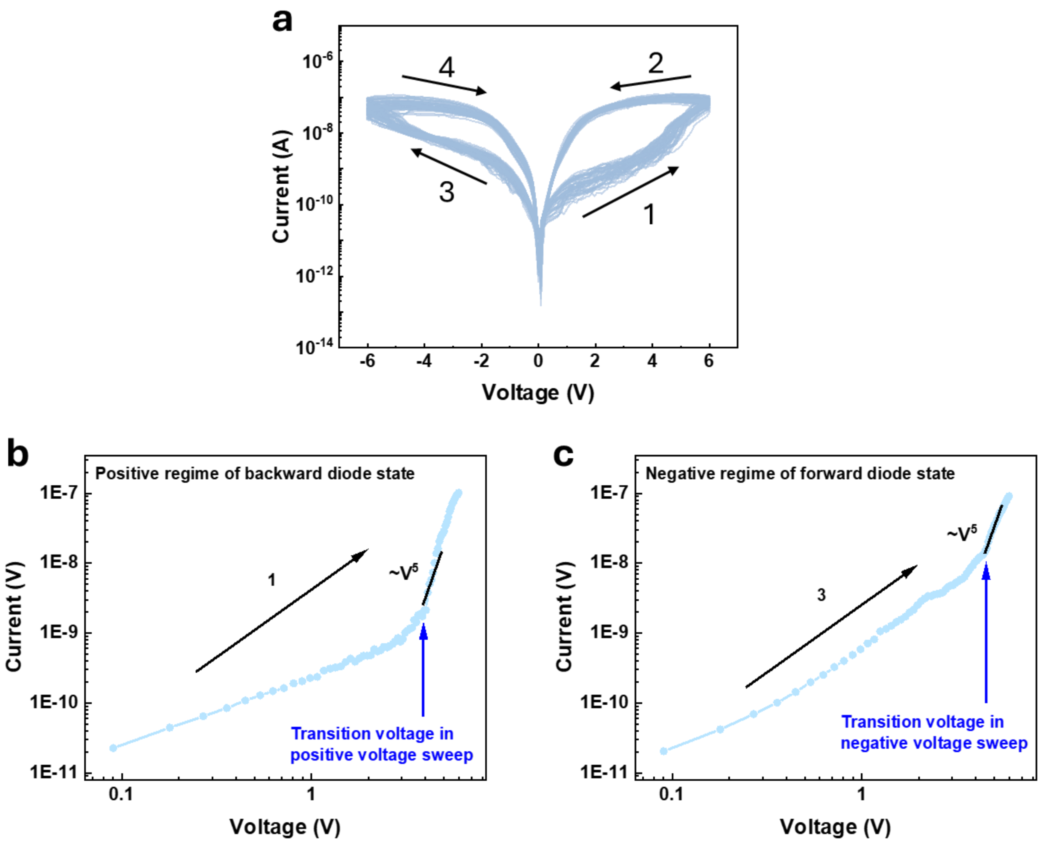

Figure S5. Transition voltage extraction in positive and negative voltage sweep. a) *I*-*V* curves of a device. The first and third segment are used for transition voltage extraction. b) Fitting in backward to forward diode state transition. Slope of the double logscale plot is fitted, and transition voltage is voltage where the nonlinearity is larger than 5 ($I \sim V^k$ and $k \geqslant 5$). c) Fitting in forward to backward diode state transition. Slope of the double log-scale plot is fitted, and transition voltage is voltage where the nonlinearity is larger than 5 ($I \sim V^k$ and $k \geqslant 5$). Transition voltage points are labelled by blue arrow.[1]

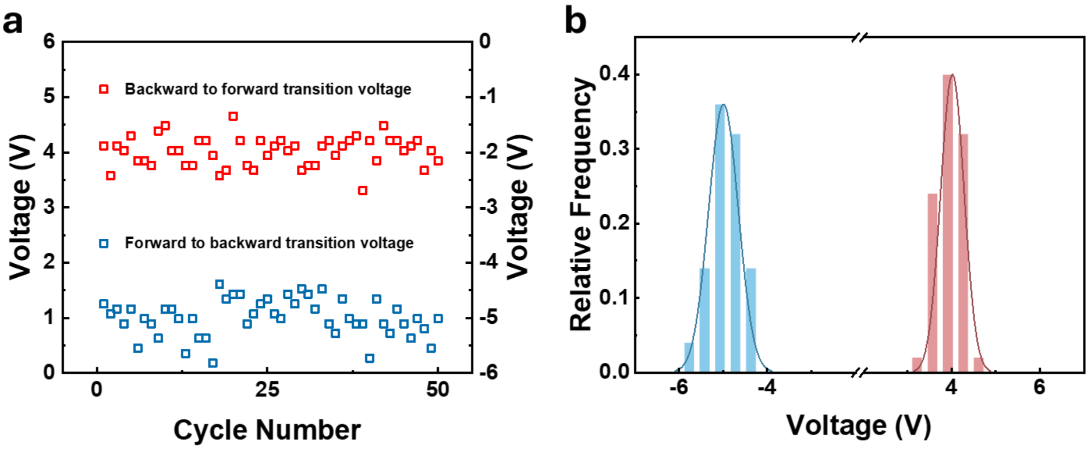

Figure S6. a) Extracted B-F and F-B transition voltages during the 50 sweeping cycles. The voltage variation is calculated by the ratio of standard deviation (σ) to average (μ). b) Histogram of the extracted B–F and F–B transition voltages, and both the distributions follow Gaussian profile.

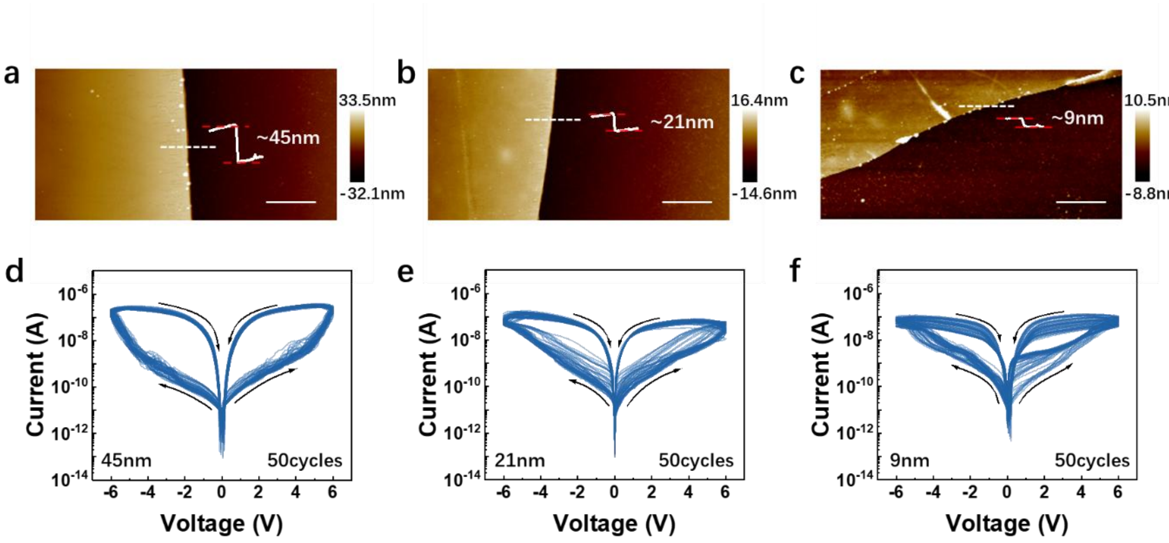

Figure S7. AFM topography image of the $NbOCl_2$ flake with various thicknesses: a) 45 nm, b) 21 nm and c) 9 nm. d-f) *I-V* curves (50 consecutive switching cycles) of c-axis devices with different channel thicknesses, corresponding to panel a-c). Scale bars are 1 μm.

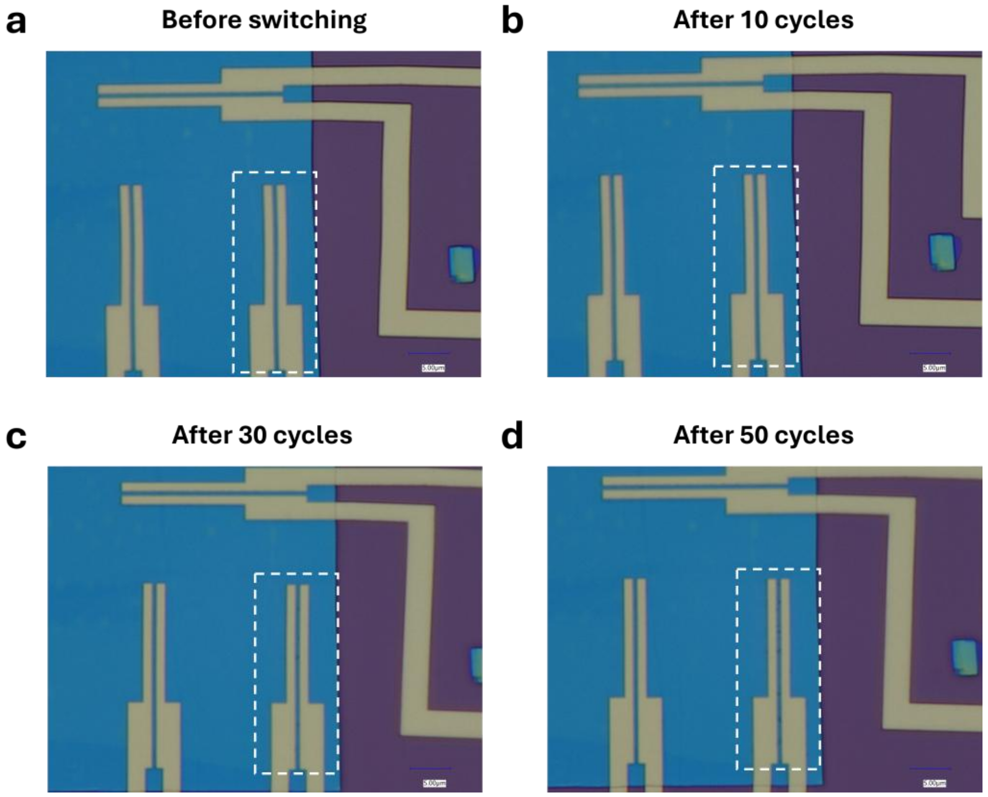

Figure S8. Original optical microscopy images corresponding to Figure 2a-2d: a) before switching, b) after 10 cycles, c) after 30 cycles and d) after 50 cycles. The measured c-axis device shown in Figure 2a-2d is highlighted by the dashed box.

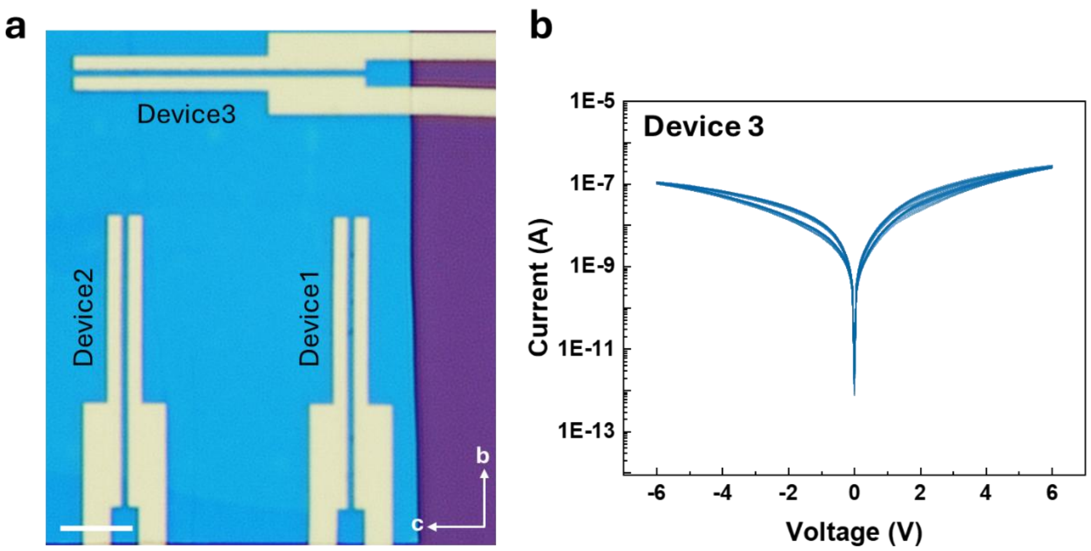

Figure S9. a) Optical image of three $NbOCl_2$ devices. Device 1 (after 30 sweeping cycles in this image) is the same device demonstrated in Figure 2a-d. Device 2 is left with no voltage applied on it. Device 3 goes through dozens of voltage sweeps. Device 1 and 2 are along c-axis, while Device 3 is along b-axis. b) *I-V* curves of Device 3 under dozens of consecutive voltage sweeps. Scale bar is 5 μm.

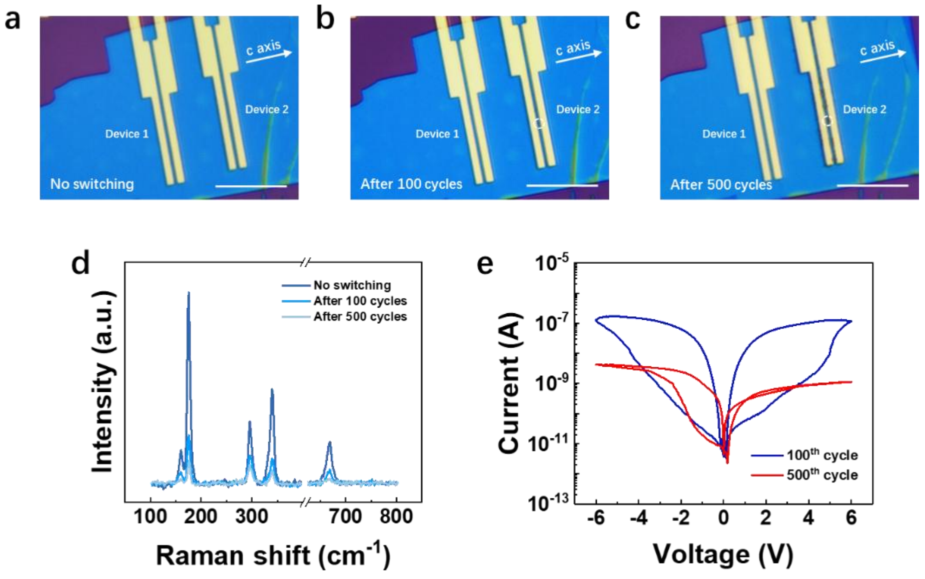

Figure S10. Optical microscopy images of the c-axis device after different switching cycles, with only Device 2 subjected to switching: a) No switching, b) After 100 cycles and c) After 500 cycles. d) Raman spectra of the circled area after various switching cycles. e) *I-V* curves of the 100th switching cycle and the 500th switching cycle. Scale bars are 10 μm.

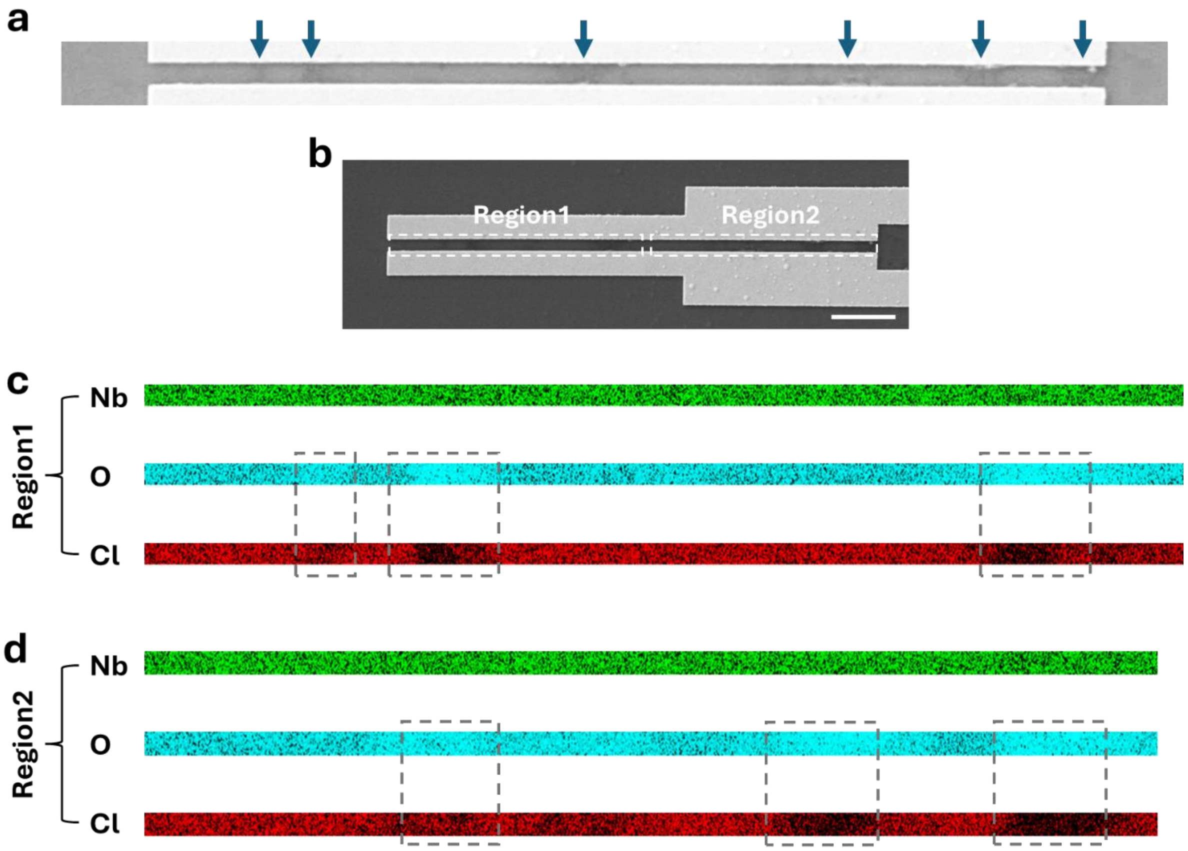

Figure S11. a) Magnified SEM image of the probed channel region in Figure 2e. Blue arrows label the darkened areas in the channel. b) The probed channel region is divided into two parts for EDS mapping. c-d) Element spatial distribution of Nb, O and Cl in two parts, respectively. Grey dash frames indicate the areas of oxidation. Scale bar is 5 μm.

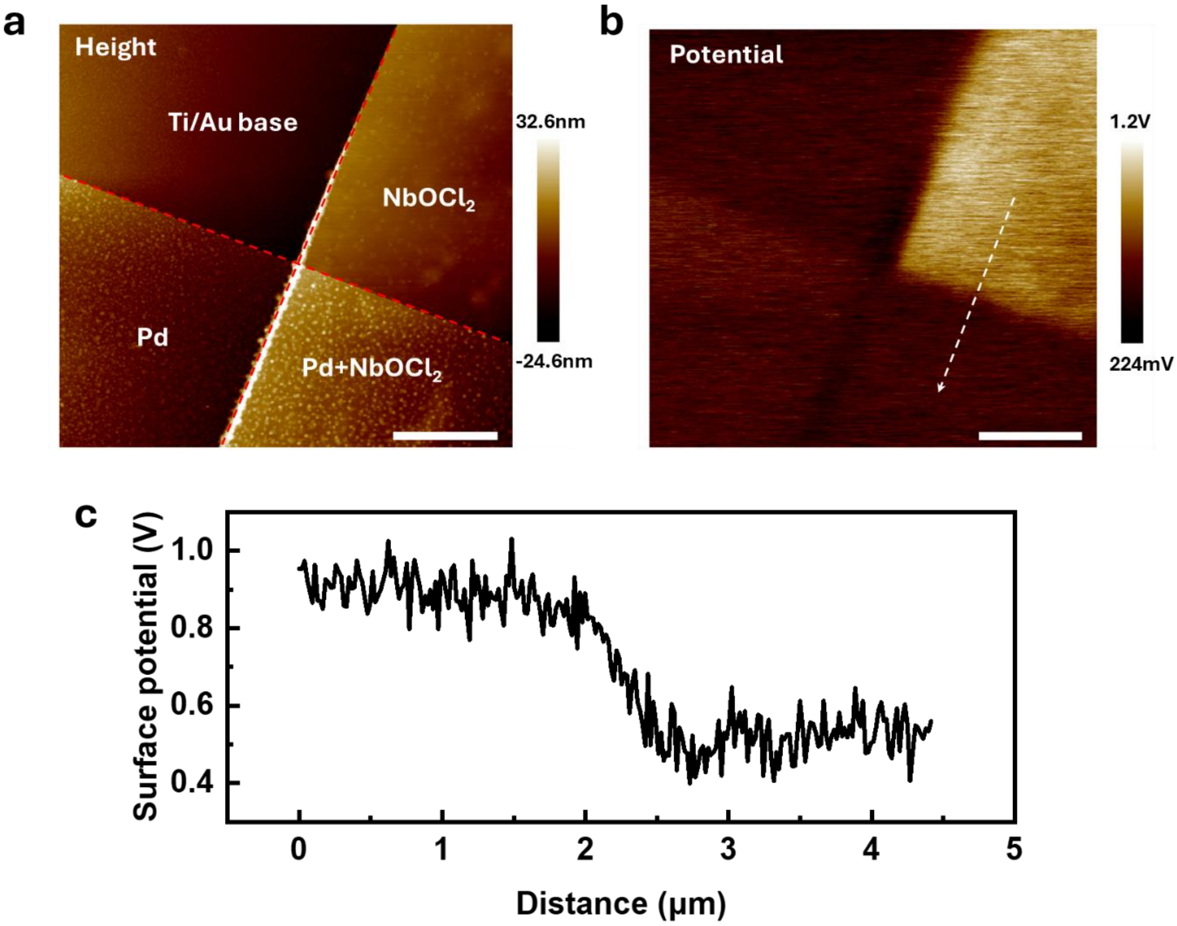

Figure S12. a) Topography image of the Pd/$NbOCl_2$/Au/Ti stack. b) Corresponding potential mapping image of the region in panel a). c) Surface potential line profile along the white dash arrow in panel b). Scale bars are 2 μm.

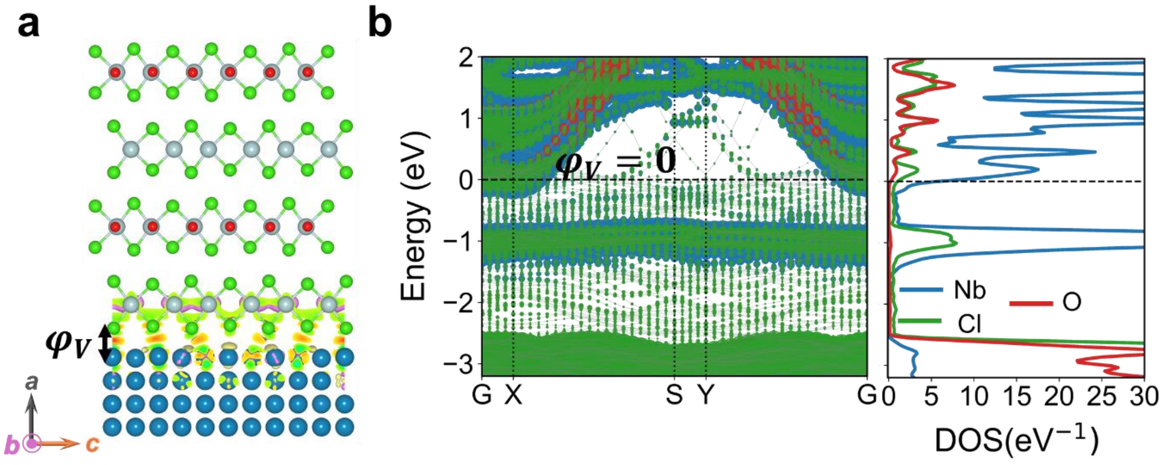

Figure S13. Electronic Properties of the vertical Pd-$NbOCl_2$ Interface. a) Structural snapshot and charge density difference isosurface plot for the vertical Pd-$NbOCl_2$ contact, highlighting the charge distribution at the interface. b) Projected band structure and density of states (DOS) of $NbOCl_2$ in Pd-$NbOCl_2$ heterostructure. The crossing of the conduction band minimum over the Fermi level indicates an ohmic behavior with a vertical Schottky barrier height (φ_V) of zero.

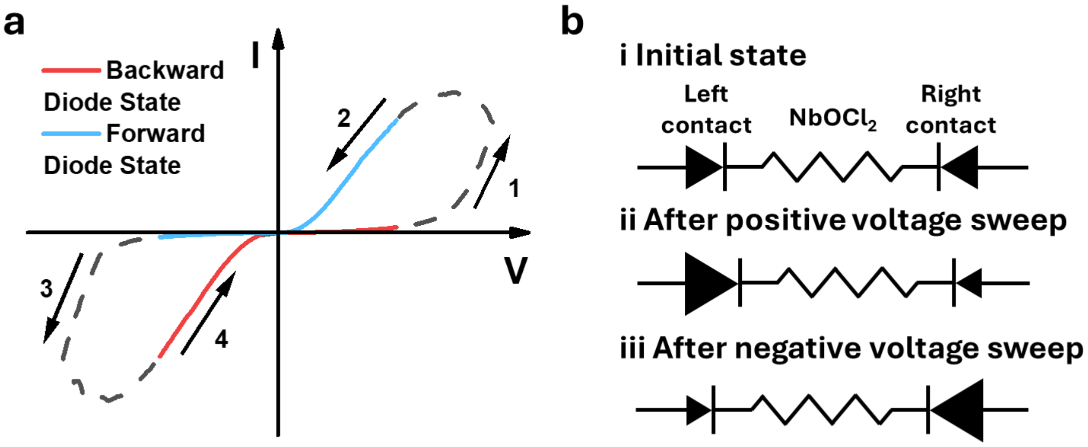

Figure S14. a) Schematic of *I-V* curve in linear scale. The black arrows indicate the voltage sweep order. The blue and red curves are two states at small voltage range after positive and negative voltage sweeps, respectively. b) Equivalent circuit models of the device for explaining the *I-V* characteristic in the initial state (i) and under certain voltages (ii and iii).

Table S1. Comparison of the energy consumption of the lateral $NbOCl_2$ memristor with the previously reported studies.

No	Structure	Energy consumption	Reference
1	$Au/MoS_2/Au$	200 pJ	[1]
2	$Ag/MoS_2/Pt$	400 nJ	[2]
3	$Ag/Ti_3C_2T_xNS/Pt$	18.82 nJ	[3]
4	$TiN/TiO_xN_y/SnO_x/Pt$	3.24 nJ	[4]
5	$Au/Li_xMoS_2/Au$	200 pJ	[5]
6	$Pt/TaO_x/TiO_y/Ti$	12.69 nJ	[6]
7	$Ag/HfO_2/C$	40 pJ	[7]
8	ITO/PEDOT: PSS/3AMPDJ/PMMA/Ag	26 nJ	[8]
9	$Ta/TaO_x/TiO_2/Ti$	6.9 nJ	[9]
10	Ag/protein nanowires/Pt	5 nJ	[10]
11	$Pd/NbOCl_2/Pd$	3.56 nJ	This work

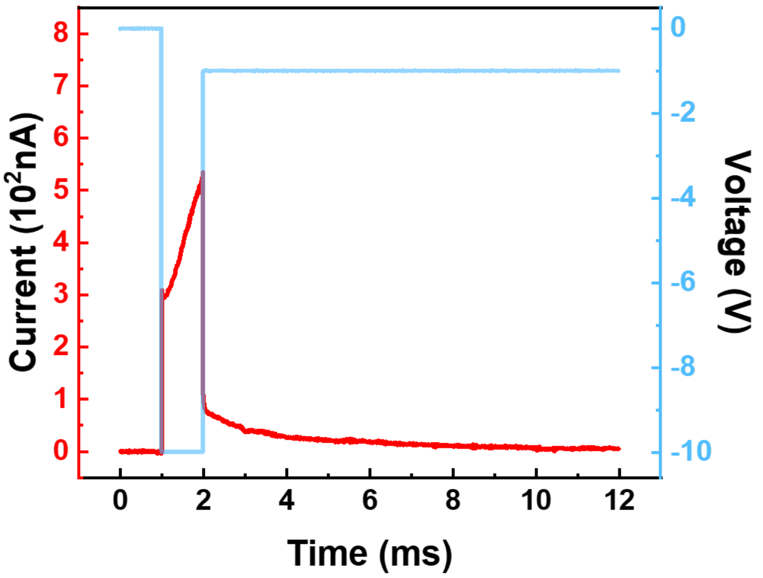

Figure S15. Relaxation of the post-pulse current (red curve) under -1 V read voltage (blue curve).

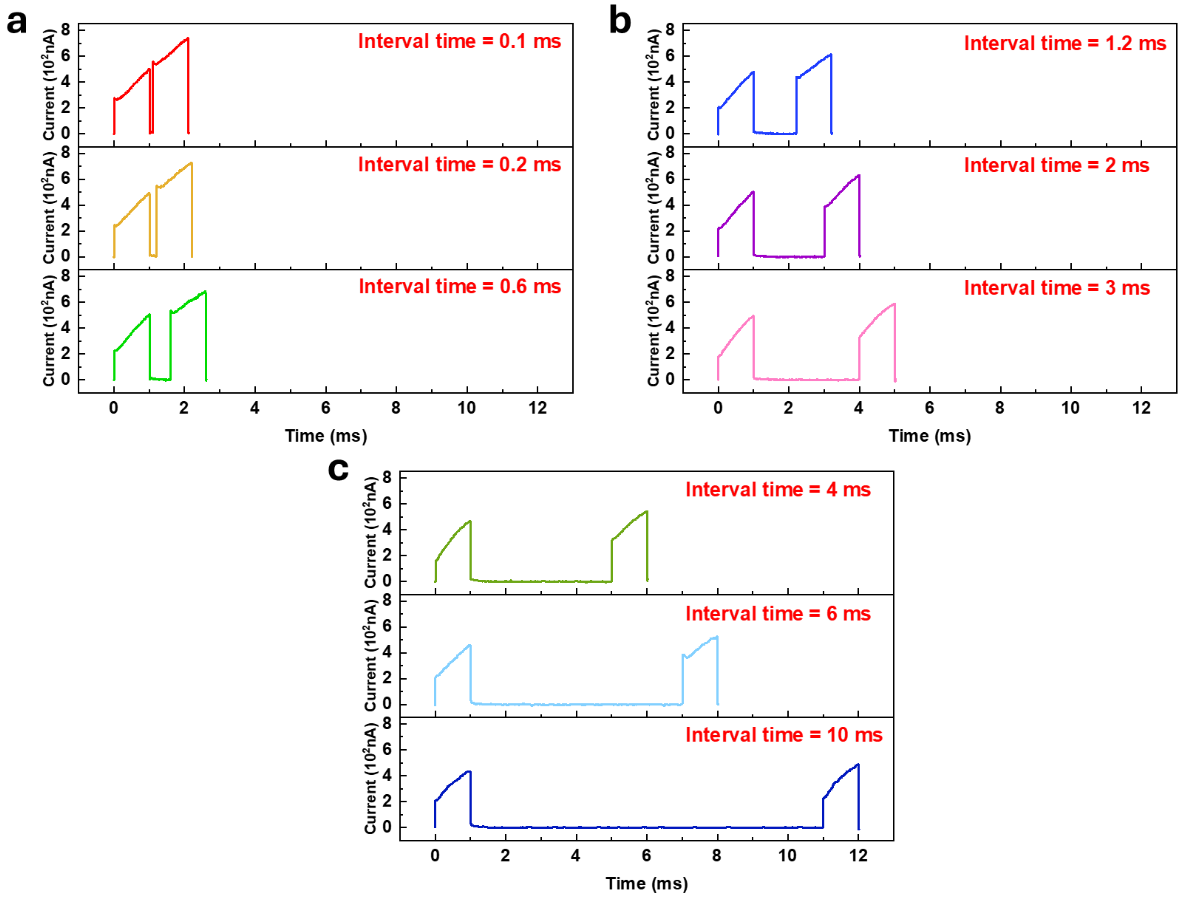

Figure S16. PPF characteristics of the $NbOCl_2$ lateral memristor. a) Interval times of 0.1 ms, 0.2 ms, and 0.6 ms, b) interval times of 1.2 ms, 2 ms, and 3 ms, and c) interval times of 4 ms, 6 ms, and 10 ms.

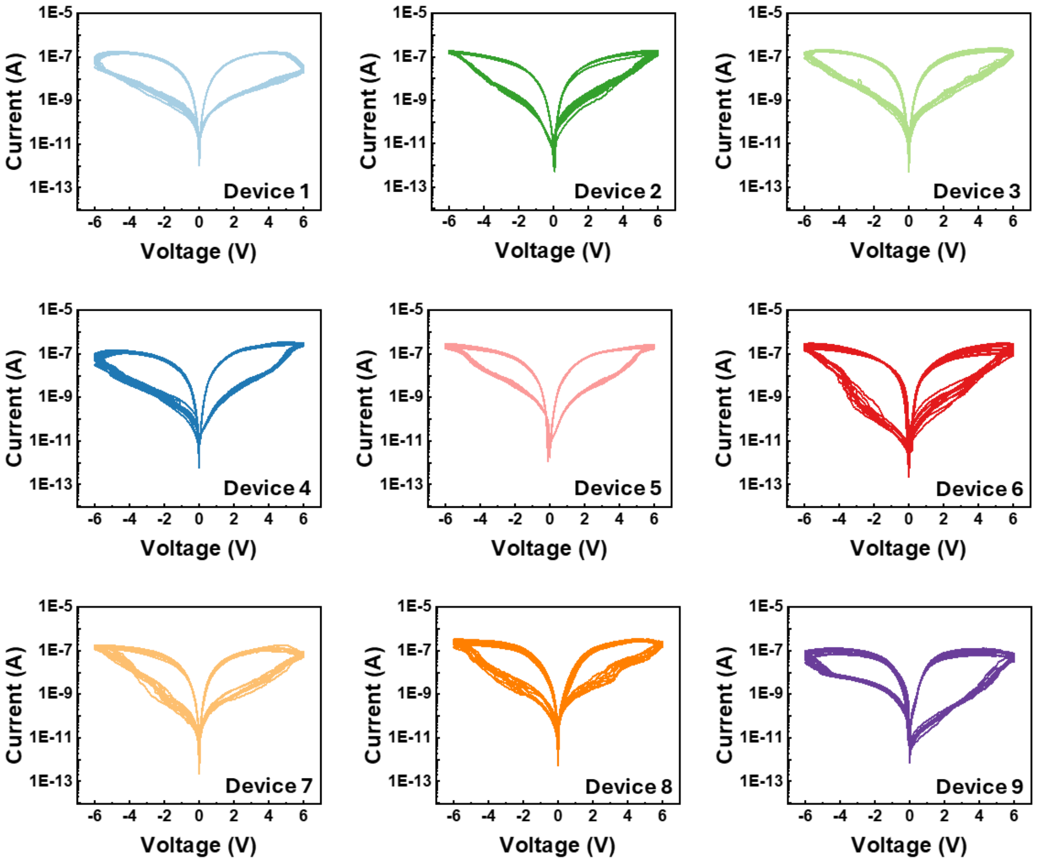

Figure S17. *I-V* curves measured over 10 cycles across 9 randomly selected $NbOCl_2$ lateral memristors.

Reference

1. E. Lee, J. Kim, J. Park, et al., "Realizing Electronic Synapses by Defect Engineering in Polycrystalline Two-Dimensional MoS_2 for Neuromorphic Computing," *ACS Applied Materials & Interfaces* (2023): 15839–47, https://doi.org/10.1021/acsami.2c21688.
2. K. Wang, L. Li, R. Zhao, et al., "A Pure 2H-MoS_2 Nanosheet-Based Memristor with Low Power Consumption and Linear Multilevel Storage for Artificial Synapse Emulator," *Advanced Electronic Materials* (2020): 1901342, https://doi.org/10.1002/aelm.201901342.
3. A. Sokolov, M. Ali, H. Li, et al., "Partially Oxidized MXene $Ti_3C_2T_x$ Sheets for Memristor having Synapse and Threshold Resistive Switching Characteristics.," *Advanced Electronic Materials* (2021): 2000866, https://doi.org/10.1002/aelm.202000866.
4. M. Ismail, D. Kim, E. Lim, et al., "Exploration of Analog Synaptic Plasticity and Convolutional Neural Network Simulation in Bilayer TiO_xN_y/SnO_x Memristor for Neuromorphic Systems," *ACS Materials Letters* (2024): 3514–22, https://doi.org/10.1021/acsmaterialslett.4c00406.
5. X. Zhu, D. Li, X. Liang, et al., "Ionic modulation and ionic coupling effects in MoS_2 devices for neuromorphic computing," *Nature Materials* (2019): 141–8, https://doi.org/10.1038/s41563-018-0248-5.
6. M. Zhu, Z. Yu, G. Hu, et al., "A TaO_x/TiO_y Bilayer Memristor with Enhanced Synaptic Features for Neuromorphic Computing," *Advanced Electronic Materials* (2024): 2400008, https://doi.org/10.1002/aelm.202400008.
7. A. Milozzi, S. Ricci, D. Ielmini. "Memristive tonotopic mapping with volatile resistive switching memory devices." *Nature Communications* (2024): 2812, https://doi.org/10.1038/s41467-024-47228-1.
8. M. Khemnani, B. Tripathi, P. Thakkar, et al., "Investigating the Role of Interfacial Layer for Resistive Switching in a Hybrid Dion-Jacobson Perovskite-Based Memristor," ACS *Applied Electronic Materials* (2023): 5249–56, https://doi.org/10.1021/acsaelm.3c01038.
9. Y. F. Wang, Y. C. Lin, I. T. Wang, et al., "Characterization and Modeling of Nonfilamentary $Ta/TaO_x/TiO_2/Ti$ Analog Synaptic Device," *Scientific Reports* (2015): 10150, https://doi.org/10.1038/srep10150.
10. S. Choi, J. Yang, G. Wang, "Emerging Memristive Artificial Synapses and Neurons for Energy-Efficient Neuromorphic Computing," *Advanced Materials* (2020): 2004659, https://doi.org/10.1002/adma.202004659.
